# Hypercalcemia-leukocytosis syndrome in a patient with cavitating squamous cell carcinoma of the lung

**DOI:** 10.1186/1757-1626-2-108

**Published:** 2009-01-31

**Authors:** Olga Burzyantseva, Sanath Dharmasena, Suriya Jayawardena, Vijay A Rupanagudi, Padmanabhan Krishnan

**Affiliations:** 1 Department of Medicine, Coney Island Hospital, 2601 Ocean Parkway, Brooklyn, NY 11235, USA; 2 Department of Cardiology, Coney Island Hospital, 2601 Ocean Parkway, Brooklyn, NY 11235, USA; 3 Department of Pulmonology, Coney Island Hospital, 2601 Ocean Parkway, Brooklyn, NY 11235, USA

## Abstract

**Introduction:**

Lung cancer is the leading cause of death among the cancers seen in the United States. Hypercalcemia and leukocytosis are two common paraneoplastic syndromes associated with lung cancer. Unfortunately patients presenting with Hypercalcemia- leukocytosis syndrome has a worse prognosis than patients presenting with lung cancer alone.

**Case presentation:**

We present a 67 yr old Caucasian male with a history of active smoking presenting as pneumonia being diagnosed as cavitating squamous cell carcinoma of the lung with hypercalcemia-leukocytosis syndrome

**Conclusion:**

There should be a high degree of suspicion to diagnose lung cancer in patients presenting with symptoms of paraneoplastic syndrome.

## Introduction

Malignant neoplasms remain the second most common cause of death in the United States, and out of these neoplasms lung cancer is the leading cause of mortality.[1] Paraneoplastic neurologic syndromes (PNS) is a rare group of disorders associated with lung cancer and believed to be due to autoimmunity against normal neuronal tissue as a result of similar neuronal antigens expressed by tumor cells. Unfortunately there is no cost effective screening method to detect lung cancer but if patients presents with features of PNS, early detection and treatment of the underlying tumor offers the best chance of treatment [2]

## Case report

A 67 year old Caucasian male presented to the emergency department with complaints of high grade fever, cough with yellowish phlegm for the past three days. He also had exertional dyspnea New York Heart Association (NYHA grade 3), which was attributed to his heart failure from under lying ischemic heart disease. The dyspnea had worsened from Grade 2 to Grade 3 over the past 5–6 months. There was no chest pain.

He had a history of atherosclerotic heart disease status post coronary artery bypass graft done three years ago and congestive heart failure (CHF), Diabetes Mellitus type II and hypertension for the past 15 years. He smoked a pack of cigarettes every day for thirty years. He was on aspirin, carvedilol, lisinopril, atorvastatin, furosemide and glipizide.

On examination, the patient was in no apparent distress. He was icteric, not cyanosed, no clubbing, no palpable lymph nodes, poor peripheral pulses and bilateral pedal edema. Jugular venous distension of 3–4 cm was noted. Auscultation of the lungs revealed decreased air entry in right lower zone and bibasilar rales. A systolic ejection murmur grade 2/6 was heard in the aortic area. Abdomen was soft with a 2 finger breadth enlargement of the liver. Rest of the examination was normal.

Complete blood count showed leukocytosis with predominant neutrophils and a left shift. Hemoglobin-hematocrit and platelet count was normal. The patient had slight hyponatremia (132 meq), hypercalcemia 13.5 mg/dl, while other serum electrolytes were within normal range. He had a raised total bilirubin (5.3 mg/dl) while other liver function tests were normal.

**Figure 1:**
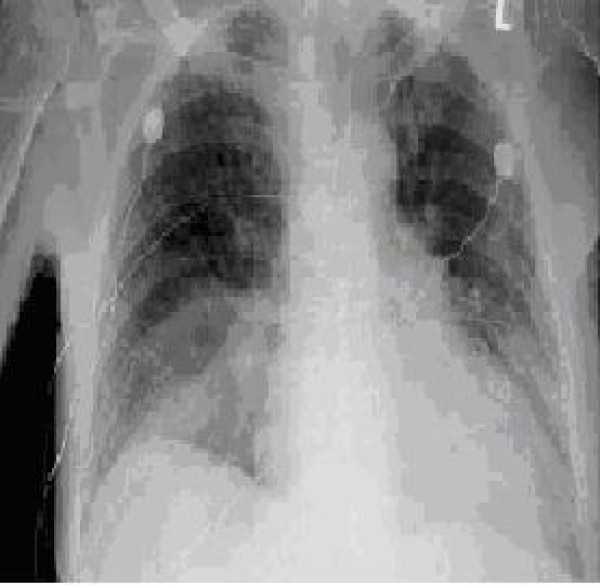
**CXR with right lower lobe infiltrate**.

Chest X-ray showed bilateral pleural effusion, right lower lung field infiltrate, cardiomegaly and chronic interstitial lung changes. The impression was right lower lobe pneumonia (figure 1). An EKG demonstrated sinus rhythm with q waves in leads II, II, aVF and V1-V5 suggestive of old infarct.

A presumptive diagnosis of pneumonia was made and he was started on intravenous ciprofloxacin, Piperacillin/Tazobactam. The increase in bilirubin was speculated to be secondary to congestion of the liver due to CHF and he was treated with furosemide, digoxin, and captopril.

**Figure 2:**
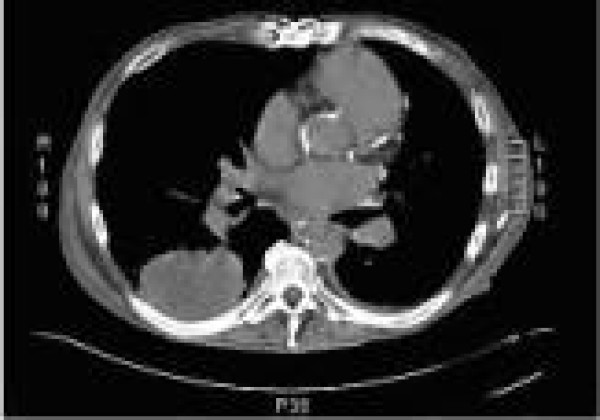
**CT chest shows pleural based lesion in the right lower lobe measuring 7 cm (SI) × 5.4 cm (AP) × 7.9 cm (TV)**.

**Figure 3:**
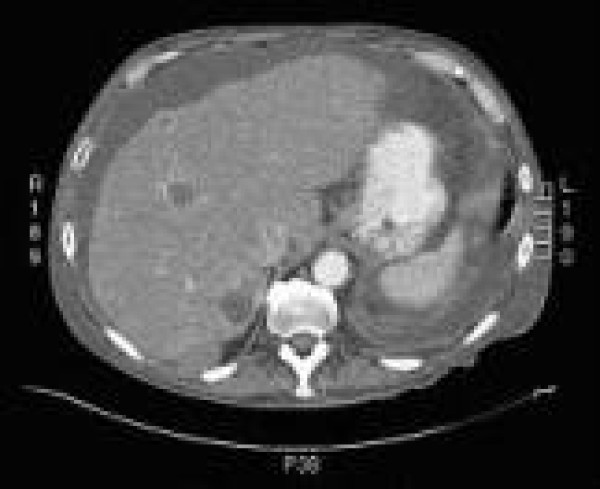
**Abdominal CT showing multiple low attenuation lesions with a peripheral rim enhancement of varying sizes in the liver suggestive of metastases or septic emboli**.

Chest CT without contrast showed a mixed density pleural based lesion in the right lower lobe measuring 7 cm (SI) × 5.4 cm (AP) × 7.9 cm (TV) for which a diagnostic consideration of cavitating neoplasm, infectious lesion and sequestration was entertained (Figure 2) There was no evidence of mediastinal, axillary or hilar adenopathy. Abdominal CT without contrast showed a large amount of intraperitoneal ascites present within abdomen and pelvis. The liver demonstrated a nodular contour suggestive of cirrhotic morphology. Multiple bilobed low attenuation lesions with a peripheral rim enhancement of varying sizes suggestive of metastases versus septic emboli were seen (Figure 3). A soft tissue mass adjacent to the posterior segment of the right lobe of the liver which could represent an exophytic liver lesion versus a mesenteric soft tissue mass was seen. A CT scan with contrast was suggested but the patient refused due to previous contrast allergy.

Fiberoptic bronchoscopy was done on the 5th days after admission and patient tolerated the procedure well. No endobronchial lesions were seen. Bronchial lavage, aspirate and brushings from the right lower lobe were done and 20 cc of hemorrhagic fluid was sent for analysis which showed few atypical squamous cells, mixed respiratory flora, no acid fast bacilli, no growth on culture. A transbronchial biopsy of the right lower lobe was done and the biopsy specimen consisted of several tan grey soft tissues measuring 1.2 × 0.2 × 0.1 cm. The biopsies showed essential bronchial mucosa with respiratory epithelium and foci of squamous metaplasia with acute inflammation.

Since fiberoptic bronchoscopy was inconclusive a CT guided transthoracic needle biopsy was done which yield 50 cc of yellow brown purulent material was aspirated from posterior pleural based collection in the right lower lobe at the same time due the questionable.

The purulent material obtained on CT guided biopsy of the lung revealed squamous cell carcinoma with extensive coagulation necrosis. There was no evidence of infection. Hence, all antibiotics were discontinued and oncology consult was obtained. CT guided needle core biopsy of the liver was negative for malignancy and showed mild inflammatory changes. Whole body PET scan was done to detect secondaries, but showed degenerative arthritic changes in knees and feet and no other focal changes that would suggest metastases. The patient had a persistent elevated leukocyte count with an elevated polymorphonuclear cell count and elevated calcium levels. The patient was offered sugary but the patient and the family refused surgery as the patients had moderate to high risk mortality from the surgery due to his multiple medical problems. The patient's poor prognosis was discussed with him and was placed in hospice care.

## Discussion

Malignant neoplasms remain the second most common cause of death in the United States, and lung cancer kills more than any other type of cancer including breast cancer, prostate cancer and colon cancer combined [1] The chest X-ray and CT scan can be used to diagnose lung cancer as in our patient but at a later stage. This delays the diagnosis leading to a guarded prognosis due to advanced disease process [3]

Can these cancers be diagnosed at an early stage? Potential molecular biomarkers for squamous cell carcinomas have been identified and need to be validated in their use in significantly reducing the lead time for diagnosis [4]

PNS are caused by the distal effects of the cancer but not due to invasion, metastases, infection, ischemia, metabolic and nutritional deficits, surgery, or other forms of tumor treatment. [5] The incidence of PNS for solid tumors is < 1%. [6] These syndromes are not frequent or pathognomonic for a signal definitive diagnosis thus there should be a high degree of clinic suspicion. There are strong association of PNS with certain malignancies, such as limbic encephalitis and subacute cerebellar degeneration. These syndromes are called classical PNS. [7] There are other syndromes, such as sensorimotor polyneuropathy may have some association with malignancies. [7]

It is believed that PNS is due to immunologic response to ectopic antigen expressed in the tumor (onconeuroal antigens) sharing the same antigens in the nervous system giving rise to cross reactivity to the antibodies produced by the body. [5]

The importance in diagnosing PNS is at the time of PNS symptoms, most patients would have no been diagnosed with cancer, thus PNS can be used as a early diagnosing tool [2] Antibodies have been used to detect PNS. These antibodies are generally divided in to three groups, first group belongs to well-characterized antibodies, are reactive with molecularly defined onconeural antigens and has a strongly association to cancer, second group is partially characterized antibodies with an unidentified target antigens which has been reported in few patients and the third group consists of antibodies associated with specific disorders but do no have the ability to differentiate between paraneoplastic and nonparaneoplastic cases [7]

Lung cancer has been associated with various hematological and laboratory abnormalities, some are part of PNS. Small cell carcinoma has been reported to be associated with marked hyperamylasemia [8] Our patient had persistent hypercalcemia and leukocytosis. Both of these have been associated with various malignancies, including lung cancer as part of the PNS either independently or very rarely together [9,10] The secretion of hematopoietic growth factors like granulocyte-macrophage colony-stimulating factor (GM-CSF), granulocyte-colony stimulating factor (G-CSF), parathyroid hormone-related peptide, interleukin (IL)-1, IL-6, and tumor necrosis factor (TNF) are activated by squamous cell carcinoma cells and their action on the common precursors of osteoclasts and granulomonocytic cells simultaneously may attribute to the presence of hypercalcemia-leukocytosis syndrome [11,12] Hypercalcemia has been shown to be an ominous prognostic sign in several malignancies [13,14] In lung cancer patients, survival of patients with the hypercalcemia-leukocytosis syndrome has been shown to be shorter than that with hypercalcemia alone [15] The presence of this hypercalcemia-leukocytosis syndrome could have been a factor in the poor prognosis of our patient.

The goal of detection of PNS is for early diagnoses of the malignancy and treatment of the tumor and the PNS. It has been shown that anti-tumor therapy is effective against tumor as well symptoms of PNS such as neurological deterioration and give the patient on average better symptom relief. [16]

Studies have shown despite the autoimmune etiology, immunotherapy has been disappointing in the treatment of PSN [16] Immunotherapy modalities currently recommended are plasma exchange, immunoadsorption (extraction of patient IgG over a protein A column), steroids, and intravenous immunoglobin [17]

## Conclusion

Lung cancer continues to present in advanced stages with metastases at the time of diagnosis making the prognosis poor. PNS associated with lung cancer if detected early can prompt early detection, early treatment and better prognosis. Unfortunately PNS is rarely seen and there should be a high degree of suspicion to diagnose PNS. In patients with PNS like in our patient the presence of leukocytosis-hypercalcemia syndrome compounds the already poor prognosis of advanced metastases.

## Abbreviations

NYHA: New York Heart Association; CHF: congestive heart failure; EKG: Electrocardiogram; Echo: Echocardiogram; GM-CSF: granulocyte-macrophage colony-stimulating factor; G-CSF: granulocyte-colony stimulating factor; IL: interleukin; TNF: tumor necrosis factor; PNS: Paraneoplastic syndrome.

## Authors' affiliations

The above case report was written at Coney Island Hospital. The above mentioned authors have no affiliation to any other institute other than Coney Island Hospital.

## Consent

A written informed consent was obtained from the patient for publication of this case report and accompanying images. A copy of the written consent will be made available on request

## Competing interests

The authors declare that they have no competing interests.

## Authors' contributions

OB, SD, SJ treated the patent and were responsible for writing the paper and looking up the back ground references.

VR, PK was responsible for over all coordination and final proof reading.

All the above mentioned authors read and approved the final manuscript.
